# Ethnic Inequalities in Psychological Distress: A Population Data Linkage Study on the Pacific Island of Guåhån/Guam

**DOI:** 10.1007/s10903-018-0815-5

**Published:** 2018-09-03

**Authors:** Tania J. Bosqui, Anne Kouvonen, Yoshito Kawabata

**Affiliations:** 1 Division of Social and Behavioural Sciences, UOG Station, University of Guam, Mangilao, Guam; 2 Faculty of Social Sciences, University of Helsinki, Helsinki, Finland; 3 SWPS University of Social Sciences and Humanities in Wroclaw, Wroclaw, Poland; 4 UKCRC Centre of Excellence for Public Health, Queen’s University Belfast, Belfast, UK; 5 Department of Psychology, American University of Beirut, P.O.Box 11-0236, Riad El-Solh, Beirut, 1107 2020 Lebanon; 6 Administrative Data Research Centre Northern Ireland (ADRC-NI), Centre for Public Health, Queen’s University Belfast, Belfast, UK

**Keywords:** Psychological distress, Mental health, Ethnicity, Guam, Inequality

## Abstract

Psychological distress and mental illness has been found to be elevated in migrant groups living in sovereign countries, as well as for indigenous people living under colonial or administrative rule. The north Pacific island of Guam is unusual in its ethnic composition as it has no majority ethnic group, has a large indigenous population and remains a territory of the U.S. This study aimed to identify ethnic differences in self-reported psychological distress between the main ethnic groups on Guam. The study uses a cross sectional design with data linkage methodology, drawing on the Guam Census and the Behavioral Risk Factor Surveillance System health survey for Guam. The results showed that the native Chamorro population had worse self-reported psychological distress (defined as a ‘mental health condition or emotional problem’) than White/Caucasians (OR 2.09, 95% CI 1.52–2.87), particularly for severe distress (OR 3.61, 95% CI 1.33–2.77). This relationship persisted even after adjusting for a wide range of socio-demographic and economic factors (OR 2.58, 95% CI 1.15–5.76). Other Pacific Islanders also had higher psychological distress compared to White/Caucasians, but this association was largely explained by the adjusted factors. The findings are discussed in terms of social and economic disadvantage for Pacific Island peoples on Guam, as well as the impact of colonial administration, disaffection, and lack of autonomy for the Chamorro of Guam. Recommendations are made to improve psychiatric treatment for these groups by considering wider socio-political factors in assessment and treatment, as well as broader implications for the national dialogue on self-determination.

## Background

Research on the mental health and wellbeing of migrants across the globe shows disproportionately high incidence of psychological distress [1], common mental health difficulties [2] and severe and enduring mental health difficulties and illnesses [3]. Second and more generations of migrants, also referred to as ethnic minorities, may have had many generations of their ancestors in their country of residence since migration, but still tend to have worse mental health and wellbeing than the majority native population [4]. For example, Puerto Ricans and Mexicans born in the USA have been found to have higher prevalence rates of major depressive disorder than the majority population [5]; and the minority Sami population of Norway reported greater psychological distress than the Norwegian majority [6].

This high level of psychological distress and mental illness amongst migrants and ethnic minority groups has been explained by an array of economic, social and psychological risk factors such as forced migration due to persecution [7], uncertain residency status [8], lack of access to services [9], exclusionist integration policy models [10], low cultural adaption and societal participation [11], poverty and debt [9]; exposure to racism and discrimination [12]; low family and community social capital [13], occupational stress [14], and neighbourhood isolation [15].

This research has, however, focused on particular migration histories and socio-political contexts, in which migrants suffer disproportionately from poverty, lack of opportunity and low social capital. Research on native or indigenous populations who have suffered imperialism, colonization, and genocide has clearly shown worse mental health for the native population compared to the settled migrant group or groups. For example, Inuit populations have been found to have nine times higher mental illness than populations of European decent in Alaska [5], in New Zealand, Cook Island Maoris were found to have the highest 12-month prevalence rate of mental disorders than any other ethnic group [16], and in Canada 13% of First Nations populations were found to suffer from psychological distress compared to 8% in the general population [17].

These differences may be understood in terms of the marginalisation, poverty and lack of opportunity experienced by migrants and ethnic minorities in sovereign states. Certainly, many colonised indigenous populations suffer disproportionately from all of these factors [18]. However, the increased mental illness and distress, as well as increased substance abuse, in these groups has to a greater extent been explained by cultural oppression, forced assimilation, and historical traumatisation across multiple generations [19, 20] as well as rapid massive structural social and geographic changes characterised by relative deprivation, powerlessness and alienation [5]. The wider context of colonialism, erosion of indigenous culture and suppression of efforts for self-determination are therefore important to understanding the increased risk of mental illness and distress of native colonised people.

The study of indigenous populations, as with migrants and ethnic minorities, is intricate in that there are more than 5000 indigenous cultures around the world [20]. The north Pacific island of Guam, or Guåhån, an unincorporated territory of the USA, is unusual in its ethnic composition in that it has no large majority. All ethnic groups account for less than 50% of the population, including the indigenous Chamorro and US settlers, largely of European descent [21]. The Chamorro population are thought to be of Mayo-Polynesian descent and the earliest inhabitants on the Island, around 2000 years ago [22]. Prior to European contact, Guamanians lived in a matrilineal society within a highly collectivistic culture [23]. The island was first colonised as an outpost by the Spanish in the mid-sixteenth Century, then by Japan and currently the U.S. As with many indigenous cultures, the traditional culture of Chamorros was systematically supressed and eroded after European contact. Chamorro religious practices and the matrilineal system have all but disappeared [22].

Many people on Guam have a strong American identity, since the unilateral decision by the U.S. Congress to make Guam an unincorporated organized territory after the island was ‘liberated’ by the U.S. from Japan in 1944 [23], and this identity is strengthened by ties with the U.S. army who provide a large amount of employment for local people. The army and naval bases take up almost half of the island, and are intrinsic to life on Guam. However, a small but substantial number of Chamorro islanders maintain a different narrative from liberation, that of coercive colonisation in which the majority of their land has been stolen for U.S. military gain, their soil poisoned by military activity, their people traumatised by active military service in wars unrelated to them, and their economy highly dependent on a foreign state, with no real access to the democratic process [24]. Guamanians do not have the right to vote in federal elections and have little say in the laws with which they are governed by.

Mental health on Guam has been under-researched [25], despite Micronesia having one of the highest rates of suicide in the world, particularly for young males. Research on suicide has shown that up to 38% of the Micronesian population have thought of completing suicide [26], with on average 14 out of 100,000 completing suicide every year, and 110 out of 100,000 for males between the ages of 15–24 [27]. Less than 40% of suicide deaths were completed by individuals with a recorded history of mental illness [27]. Research on suicide does little therefore to help estimate the level of psychological distress on the island. The only published quantitative study known to the authors on mental health and distress on Guam indicates a prevalence rate of 17.8% for moderate to severe depression and 40.2% for anxiety, although this research is limited by using a relatively small sample of college students, with Pacific Islanders amalgamated into one category preventing any inter-group comparisons [28]. A current study underway at Guam Behavioral Health and Wellness Centre (GBHWC), the only public psychiatric hospital on the island, involves the collection of data on all psychiatric disorders, as well as ethnicity and individual socio-economic characteristics [29] which will help to estimate prevalence rates of diagnosed psychiatric disorders and their risk factors. Wider research on Pacific Islanders living in the U.S. has found higher levels of depression amongst Pacific Islanders than the general population, but this was largely explained by discrimination, ethnic marginalization and acculturation stress all of which are associated with migration and minority status in the U.S [30]. Research on the Pacific region has also highlighted high levels of psychological distress associated with social exclusion, unemployment and limited opportunity [31] and poor mental health compared to other parts of the world, as well as poorer access to mental health services [32].

Given the lack of research on psychological distress on Guam, and the international bias that neglects the diversity of ethnic population compositions outside of Western sovereign states, their different migration histories, cultural practices and psychosocial stressors, research on ethnic differences in mental illness and psychological distress on Guam is needed. Such research is important to understand the experience of ethnic minority groups, native people and migrants, and the risk and resilience factors that affect their wellbeing. As well as the lack of prior research and unique context, Guam is also well placed for health research given its fairly well established Census and Public Health Department Surveys that provide good quality data on a range of health, social and economic factors. This study therefore aims to fill a gap in international research by using a cross-sectional data linkage methodology to obtain estimated levels of self-reported psychological distress across the main ethnic groups on Guam and to identify the relative socio-demographic and economic risk factors.

Based on previous research and available data, the study proposes the following hypotheses (1) Chamorro and other Pacific Islanders will have a higher likelihood of psychological distress than other ethnic groups living on Guam; and (2) this difference will be explained to a large extent by different socio-demographic and economic factors (including employment and wealth, substance use, access to health care, help seeking, active military experience and neighbourhood deprivation).

## Method

### Participants

This study used data from respondents of the 2010 Census Island Areas (Guam) and the Behavioral Risk Factor Surveillance System (BRFSS) from the U.S.-based Centre for Disease Control and Prevention (CDC). The total sample was made up of 2518 participants, 798 of whom reported having a mental health condition for at least 1 day in the last month. The largest ethnic groups included were Chamorro (n = 1101) and Filipino (n = 734). Details of each included ethnic category are displayed in Table 1.

**Table 1:** Sociodemographic participant characteristics, by ethnicity

	Ethnicity (total n = 2518)				
	Chamorro (n = 1101)	Other Pacific Islanders^a^ (n = 222)	Filipino (n = 734)	White/ Caucasian (n = 259)	Other^b^ (n = 202)
Psychological distress (%)					
Yes	37.60	32.88	25.89	22.39	31.19
Socio-demographic characteristics					
Female (%)	58.95	57.66	58.45	40.15	50.50
Age (M, SD)	43.63 (16.19)	36.59 (12.74)	46.07 (16.38)	45.56 (17.95)	35.72 (15.73)
Marital status (%)^c^					
Married	47.32	36.04	61.85	65.64	59.41
Other	52.68	63.96	38.15	34.36	40.59
Highest completed education (%)					
High school or less	57.22	62.16	33.79	23.17	31.68
More than high school	42.78	37.84	66.21	76.83	68.32
Employment (%)^d^					
Employed	54.40	51.35	62.94	57.92	63.37
Self-employed	5.18	4.95	4.09	9.65	10.40
Other	40.42	43.42	32.97	32.43	26.24
Income (%)^e^					
Less than $15,000	13.08	33.78	14.58	4.63	8.92
$15,000–$24,999	17.26	18.02	23.43	7.72	11.88
$25,000–$34,999	11.53	12.16	14.71	10.81	10.89
$35,000–$49,999	15.89	11.26	12.67	16.22	18.81
$50,000–$74,999	5.63	11.26	7.49	4.25	5.45
$75,000 or more	19.44	6.76	10.63	36.29	25.74
Home ownership (%)^e^					
Own home	59.04	19.37	45.91	34.75	35.15
Rent	22.98	58.11	33.92	45.56	41.09
Substance use (%)					
Alcohol at least once a month	44.23	31.53	32.42	59.85	51.98
Betel nut chewing at least some days	52.68	61.26	9.40	19.30	10.40
Smokes nicotine	50.41	28.38	25.07	38.61	32.18
Smokes marijuana	41.78	30.63	14.71	28.96	21.78
Uses other illicit drug	15.08	12.16	5.04	12.74	8.42
Health care access (%)					
Has health care coverage	84.29	59.46	78.61	92.66	78.71
Not accessed healthcare due to cost	19.53	33.78	20.57	6.56	13.86
Believe or strongly believe treatment can help mental ill health	71.48	59.01	70.57	78.38	64.36
Active military service (%)	15.71	7.66	6.81	42.08	23.76
High area deprivation (%)	40.24	48.24	57.08	23.94	35.64

P < 0.001 in all cases (χ^2^ test)

^a^Includes Chuukese, Pohnpeian, Kosraean, Yapese, Palauan, Marshallese, Saipanese, Rotanese and other Pacific Islander

^b^Includes Chinese, Korean, Japanese, Black, other, refused and not sure

^c^Other includes divorced, separated, widowed, member of unmarried couple, refused and not sure

^d^Other includes out of work, homemaker, student, retired, unable to work and refused

^e^Omitted not sure and refused

### Measures

The Guam Census is based on residency on April 1, 2010 and contains 75 questions derived from the American Community Survey form. The entire enumerated population of Guam is 159,358. Due to poor mailing infrastructure, Census completion was conducted through a labour intensive list/enumerate methodology (for detail on the Guam Census methodology see Reyes and Caldwell [33]). As there is no current procedure for individual data linkage using zip codes, only publicly available neighbourhood Census data was used in this study, and linked to Census data using village names. The data was available in the Guam Statistical Yearbook, published by and obtained from the Bureau of Statistics and Plans, Hagåtña [34].

The BRFSS dataset is derived from health survey data from across the U.S. and its unincorporated territories of over 400,000 people (≥ 18), with 2518 of them on Guam (1.58% of the total population). The BRFSS is the largest current public health survey in the world (for detail on the BRFSS methodology see CDC [35]). The Guam dataset in use in this study was obtained from the Department of Public Health & Social Services, Mangilao, Guam. The majority (98.3%) of the data were collected in 2014 and the rest in 2015. Along with the core CDC questionnaire, it includes additional questions on locally relevant ethnic groups, village of residence, and Betel nut use (an Areca nut widely chewed within the western Pacific region, and with strong evidence of damaging effects on health including acute psychotic episodes [36]).

Variables derived from questions included in the Guam BRFSS (available from the authors on request) included psychological distress, ethnicity, socio-demographic and economic factors, substance use, health care access and beliefs about efficacy as well as active military service. Psychological distress is defined in the Guam BRFSS survey as a ‘mental health condition or emotional problem’ and was coded into a binary variable where ‘yes’ indicates that that the individual experienced psychological distress for at least 1 day in the last 30 days, and ‘no’ indicates that they were not affected at all. For analysis purposes, those responding ‘don’t know/not sure’ and ‘refused’ where amalgamated with ‘no’ (n = 21). For post-hoc sensitivity analysis, a psychological distress severity variable was also generated, with severe distress (20–30 days), moderate (10–19 days) and mild (1–9 days) groupings. The Guam BRFSS dataset includes 17 locally relevant ethnicity categories, although some ethnic groups had small sample sizes and had to be grouped into larger categories. The ethnicity terminology used in this study is taken verbatim from the categories in the BRFSS. Care was taken in grouping self-reported ethnic categories due to the acknowledged limitations of socially constructed conceptualisations of ethnicity [4]. A priori power calculation using Demindenko’s [37] test procedure with variance correction (95% CI, α error prob 0.05, expected odds ratio of 1.5) estimated a required sample size of 417. Chamorro, Korean and White/Caucasian were all large enough to remain as distinct categories. Marshallese, Kosrean, Pohnpaian, Yapese, Saipanese, Palauan and Chuukese were amalgamated into ‘Other Pacific Islander’ and Chinese, Korean, Japanese and ‘Black’ were amalgamated with ‘Other.’

Gender (male and female) and age (18–29, 30–39, 40–49, 50–59, 60–69, 70–79 and 80 or over) were coded as categorical variables with no missing data. Socio-economic characteristics included marital status, highest educational attainment, employment status, home ownership, and income. Substance use included ratings of alcohol use, chewing of betel nut, smoking cigarettes, marijuana use and other illicit drug use. Alcohol use was coded as ‘at least once a week’, ‘at least once a month’ or ‘none,’ all other substances were codes as ‘yes’ (any use within the last month) and ‘no’ (not used in the last month). Level of health care access incorporated both health care coverage and whether health services could not be accessed due to cost in the last year. Beliefs about healthcare contained one question asking the level of agreement about whether or not treatment can help people with mental illness lead normal lives. Finally, military service included one question on whether or not the person has actively served in the United States Armed Forces. Unless otherwise specified, all missing data was amalgamated with the categories of ‘don’t know/not sure’ and ‘refused,’ due to low cell count, and was included in the analyses as a separate category. It was ensured that data presented in raw form, such as in Table 1, was non-identifiable by amalgamating or omitting categories with any call count less than 10 (as per the best practice guidelines of ADRN [38]).

Guam Census data on Village deprivation were converted into tertiles, so that each village could be coded as low, medium or high (to ensure non-identifiability as per the guidelines of ADRN [38]; and McNabb, Timmons, Song & Puckett [39]). There are a total of 19 Villages on Guam. Villages have between 782 (Humåtak) and 20,539 (Yigu) people, range from 19.4% (Tumuning) to 87.6% (Malesso’) Chamorro population, and from 12.2% (Santa Rita) to 25.8% (Humåtak) households living below the poverty line. On Guam, poverty is defined as when ‘a family’s total income is less than that family’s threshold’ [34, p. 485].

The use of publicly available data did not require ethical approval, however data linkage did undergo the required ethical scrutiny by both the University of Guam and the Department for Public Health & Social Services. This study was therefore conducted in accordance with the ethical standards laid down in the 1964 Declaration of Helsinki and its later amendments.

### Analysis

Data were analysed using Stata 13 [40]. Stepwise multi-level logistic regression models were conducted, with psychological distress as the dependant variable, and ethnicity as the primary independent variable, providing odds ratios and their 95% confidence intervals. White/Caucasian were used as the reference group as they reported the lowest psychological distress in all the groups studied. Specification errors were first ruled out using a linktest (hatsq z = − 0.32, p = 0.747). The first model was unadjusted, second adjusted for socio-demographic characteristics, the third for substance use, the fourth for health care access and beliefs, the fifth for active military experience, the sixth for area deprivation and the final multi-level model adjusted for area deprivation, with village of residence inputted as the second level. Statistically non-significant (p > 0.05) predictors were dropped at each stage. A post-hoc sensitivity analysis was conducted using a multinomial logistic regression including the severity index for psychological distress. Relative Risk Ratios (RRRs) were produced at 95% Confidence Intervals. The model was run unadjusted and then adjusted for all included variables in the original fully adjusted logistic regression model described above.

## Results

### Participant Socio-demographic and Economic Differences

Other Pacific Islanders had the highest level of unemployment, the lowest proportion of college graduates and the lowest income of all ethnic groups, as displayed in Table 1. The Chamorro population followed the Pacific Islander profile closely, with 43% completing more than high school compared to 77% of White/Caucasians and 66% of Filipinos, and 40% not in employment. White/Caucasian participants had the lowest level of unemployment of all ethnic groups, and over a third of White/Caucasians earned over $75,000 compared to just under 7% of other Pacific Islanders. Filipino participants followed a similar profile, with the highest employment rate of 63%. However, they were the most likely (57%) to live in a high deprivation area.

### Differences in the Likelihood of Psychological Distress in Different Ethnic Groups

Table 2 provides odds ratios and CIs (95%) for the stepwise logistic regressions. The unadjusted model showed that Chamorro’s were twice as likely to report psychological distress as White/Caucasians, whilst other Pacific Islanders were 70% more likely. There was no difference between White/Caucasians and Filipinos. However, the adjusted models show Chamorro’s maintained a 50% increased likelihood but other Pacific Islanders had no difference with White/Caucasians.

**Table 2:** Stepwise logistic regression models providing odds ratios for psychological distress, by ethnicity

Reference group: White/Caucasian (reporting psychological distress n = 58)	Ethnicity (total reporting psychological distress n = 798)					
	Chamorro (reporting psychological distress n = 414)		Other Pacific Islanders^a^ (reporting psychological distress n = 73)		Filipino (reporting psychological distress n = 190)	
	Odds ratio	95% CI	Odds ratio	95% CI	Odds ratio	95% CI
Unadjusted model	2.09	1.52–2.87	1.70	1.13–2.54	1.21	0.87–1.69
Model 2 adjusting for socio-demographic characteristics^b^	1.82	1.32–2.51	1.40	0.93–2.12	1.13	0.81–1.59
Model 3 adjusting for substance use^c^	1.71	1.23–2.37	1.42	0.93–2.15	1.26	0.89–1.78
Model 4 adjusting for health care access and help seeking^d^	1.53	1.10–2.14	1.10	0.71–1.69	1.08	0.76–1.54
Fully adjusted multi-level model	1.53	1.10–2.14	1.10	0.71–1.69	1.08	0.78–1.54

^a^Includes Chuukese, Pohnpeian, Kosraean, Yapese, Palauan, Marshallese, Saipanese, Rotanese and other Pacific Islander

^b^Significant (p < 0.05) socio-demographic characteristics kept in the model are sex, marital status, and employment

^c^Significant (p < 0.05) substance use predictors kept in the model are alcohol and marihuana smoking

^d^Significant (p < 0.05) health care access and help seeking predictors kept in the model are cost of healthcare as a barrier to use and beliefs about treatment efficacy

### Differences in Psychological Distress Severity in Different Ethnic Groups

Multinomial logistic regressions to compare ethnic differences between mild, moderate and severe self-reported psychological distress is displayed in Table 3. The results show that Chamorro’s have an almost four times greater risk of psychological distress compared to White/Caucasians, compared to 90% higher risk in the mild category. Similarly, other Pacific Islanders have an almost 3 times increased risk in the severe category (p < 0.05), compared to 60% in the mild category. Similarly to the binary model, Filipinos showed no difference in the risk of psychological distress compared to White/Caucasians. The fully adjusted model, showed a decrease in this difference for all ethnic groups. However, Chamorros maintained a 2.5 times greater risk in the severe category.

**Table 3:** Multinomial logistic regression models providing Relative Risk Ratios for the severity of psychological distress, by ethnicity

Reference group: White/Caucasian (reporting psychological distress n = 58)	Ethnicity (total reporting psychological distress n = 798)					
	Chamorro (reporting psychological distress n = 414)		Other Pacific Islanders^a^ (reporting psychological distress n = 73)		Filipino (reporting psychological distress n = 190)	
	RRR	95% CI	RRR	95% CI	RRR	95% CI
Unadjusted model						
Mild (1–9 days)	1.92	1.33–2.77	1.60	0.99–2.57	1.25	0.84–1.84
Moderate (10–19)	1.79	0.93–3.45	1.49	0.64–3.46	0.84	0.41–1.74
Severe (20–30)	3.61	1.64–7.93	2.73	1.64–6.92	1.58	0.68–3.66
Fully adjusted model^b^						
Mild (1–9 days)	1.42	0.97–2.08	1.05	0.64–1.73	1.08	0.72–1.63
Moderate (10–19)	1.29	0.65–2.56	0.90	0.37–2.19	0.74	0.35–1.58
Severe (20–30)	2.58	1.15–5.76	1.74	0.66–4.58	1.47	0.62–3.47

^a^Includes Chuukese, Pohnpeian, Kosraean, Yapese, Palauan, Marshallese, Saipanese, Rotanese and other Pacific Islander

^b^Adjusted for gender, marital status, employment, alcohol use, marijuana use, unable to access healthcare due to cost, beliefs about mental health treatment

## Discussion

This study compared inter-ethnic group differences in self-reported psychological distress using data linkage methodology, the first study of its kind on Guam. The results clearly show higher levels of poverty and disadvantage amongst Chamorro and other Pacific Islanders on Guam compared to the other included ethnic groups. The results also show higher distress amongst Chamorro and other Pacific Islanders compared to White/Caucasians, who had the lowest likelihood of psychological distress. This difference was particularly pronounced in the severe end of the distress spectrum. Namely, Chamorro people were found to have an almost 4 times higher likelihood of severe psychological distress than their White/Caucasian counterparts, and other Pacific Islanders had an almost three times higher risk. This therefore supports the first hypothesis of the study, based on previous research showing poorer mental health and wellbeing amongst native peoples under colonial rule [41]. This highlights similarities between the native people of Guam and other colonised indigenous groups across the world.

After controlling for socio-demographic and economic factors, the likelihood of psychological distress for other Pacific Islanders was not statistically different from White/Caucasians on Guam, in line with past research [3] and supporting this study’s second hypothesis. This finding reflects the well-established link between socio-demographic and economic disadvantage with mental illness and distress, and indicates a need for greater societal equality on Guam. To some extent, the Chamorro population followed the same pattern described for other Pacific Islanders. A large extent of the higher likelihood of psychological distress for Chamorro participants was explained by the socio-demographic and economic factors described above. However, unlike other Pacific Islanders, the Chamorro group maintained a 50% higher likelihood of psychological distress compared to White/Caucasians, and had a 2.5 times higher likelihood of severe psychological distress, after adjusting for social disadvantage. This is contrary to the hypothesis made in this study; the mental health inequity for Chamorro people is not fully explained by socio-economic disadvantage. This is a particularly interesting result indicating that there are differences for the native Chamorro population compared to other native Pacific people on Guam that contribute to disproportionate severe psychological distress; differences that are likely to be key in tackling mental health inequalities.

Differences between indigenous populations living on their native land and those who have migrated have been found in past research. In New Zealand, the Te Rau Hinengaro study found that although Cook Island Maori and other Pacific peoples had higher mental illness and distress than the rest of the New Zealand population, there were major inter-group differences between Pacific peoples who were New Zealand born and those who had migrated after 18 years of age. The former group had a 12 month prevalence rate of 31.4%, compared to only 15% in the latter [16]. This is unlikely to be explained by migration itself, given the detrimental effect of migration on mental health found in other parts of the world (after the ‘healthy migrant effect’ wears off rapidly after migration [42]). Furthermore, a study in Canada found inter-group differences in rates of suicides within indigenous communities born in British Colombia: suicide was dependent on the extent to which the community had local control, including self-government, control of health service provision and cultural facilities [17].

These previous findings have been explained by the psychological impact of colonial rule, erosion of identity and lack of autonomy, whilst living on native land [20]. This may apply to the present findings for the Chamorro people of Guam. Many other Pacific islands, from which participants in this study have migrated from, have a violent colonial history, but many have also achieved independence. Pohnpei, Yap and Chuuk represent the largest proportion of individuals in the ‘other Pacific Islander’ category in this study, making up 56.3% of the sample. These islands gained independence from the U.S. as part of the Federal States of Micronesia in 1986 after 101 years of colonial rule [43]. Smaller categories of included groups include those from Palau and Saipan, making up a further 30.2% of the ‘other Pacific Islander’ category. These islands, with 120 and 440 years of colonial rule respectively, are sovereign states with either commonwealth or free association status maintained with the U.S [43]. In line with Kirmayer et al.’s [20] argument, this may be the key difference between the two populations that explains why socio-demographic and economic disadvantage alone explains the disproportionate psychological distress in non-Chamorro Pacific Islanders but not the native Chamorro. More research on neighbouring islands is needed to confirm whether socio-political differences can explain the inter-group psychological distress inequality found in this study.

## Strengths and Limitations

The present study is the first to systematically and quantitatively study ethnic differences in psychological distress on Guam. It provides initial estimates of inter-group differences in this region, which has been neglected in past research [17]. The study draws on survey and Census data using random sampling and data linkage methodology, resulting in a large representative sample, as well as benefiting from detailed survey questions that allowed the adjustment for multiple contributory factors. Its main limitation however, is the use of a single self-report measure of psychological distress. Single-item measures of mental health are increasingly used in epidemiological research, and have evidence of convergent validity with established mental health scales [44], but are limited in assessing the complexities of mental health and wellbeing and carry an increased risk of false positive and negatives than multi-item measures. The single item question used in this study is also highly inclusive, citing both ‘emotional problem’ and ‘mental health condition.’ This could include people experiencing bereavement or interpersonal stress within normal range. It is improbable therefore that the results of this study reflect the prevalence of diagnosable psychiatric conditions but rather give an initial indication as to the level of general psychological distress in the Guam population, its contributory factors and inter-group differences. Further, the meaning of a ‘mental health condition or emotional problem’ may differ between individuals and across ethnic groups, particularly given cultural differences in taboos and stigma in talking about mental health [32] as well as conceptual differences in defining distress and ill health [5]. The lack of research using culturally validated instruments leading to only rough estimates of prevalence rates has been well highlighted in past research [5, 17], and this study is no exception. Further research using locally adapted diagnostic interviews, such as that being conducted by Miller and colleagues [29] in GBHWC will help to address this limitation. Finally, the omission of the CDC supplementary question on the exposure and emotional impact of racism by the Guam Department of Public Health & Social Services is unfortunate as this is a well-established contributory factor for psychological distress and mental illness in marginalised groups [15]. We strongly recommend that the question be included in future surveys.

## Implications and Conclusion

This study clearly demonstrates inequalities in psychological distress between ethnic groups on Guam, with Chamorro and other Pacific Islanders suffering disproportionately compared to White/Caucasians and Filipinos. This inequality is largely explained by socio-demographic and economic differences, although the impact of colonialism, identity erosion and disaffection are also likely to play a role for Chamorro people. These findings highlight the importance of social and political change on Guam to reduce mental distress and improve wellbeing for local native people. At a health care level, psychiatric treatment for Chamorro and other Pacific Islanders would do well to consider wider societal injustice in assessments and formulations, rather than solely focusing on organic, individual or familial factors. This is particularly important to prevent the pathologisation of normal reactions to oppression and disaffection in indigenous groups [45], to incorporate wider socially and culturally constructed understandings of mental distress [46], to acknowledge the impact of negative ethnic identities on wellbeing [47], and reflect the complexity of individual and socio-cultural level interactions in explaining distress.

At a wider socio-political level, achieving political recognition for indigenous people through structural socio-political change is likely to be important for the wellbeing of Chamorro people, as well as the more tangible reduction of poverty and disadvantage. Kirmayer et al. [20] argue that reducing health disparities and disadvantage, in line with the UN Sustainable Development Goals, requires legislation and policies which address specific social determinants, which in the context of indigenous peoples includes incorporating wider socio- historical and political injustices. Although Guam has an established mental health policy including prevention, treatment and rehabilitation administered by the GBHWC [48], albeit with its challenges and limitations at primary care level [32], the findings of this study indicate a need for broader socio-political change on Guam if its native population are to achieve improved and equal mental health and wellbeing.
